# Assessing the Evolutionary Impact of Amino Acid Mutations in the Human Genome

**DOI:** 10.1371/journal.pgen.1000083

**Published:** 2008-05-30

**Authors:** Adam R. Boyko, Scott H. Williamson, Amit R. Indap, Jeremiah D. Degenhardt, Ryan D. Hernandez, Kirk E. Lohmueller, Mark D. Adams, Steffen Schmidt, John J. Sninsky, Shamil R. Sunyaev, Thomas J. White, Rasmus Nielsen, Andrew G. Clark, Carlos D. Bustamante

**Affiliations:** 1 Department of Biological Statistics and Computational Biology, Cornell University, Ithaca, New York, United States of America; 2 Department of Molecular Biology and Genetics, Cornell University, Ithaca, New York, United States of America; 3 Department of Genetics, BRB-624, Case Western Reserve University, Cleveland, Ohio, United States of America; 4 Division of Genetics, Department of Medicine, Brigham & Women's Hospital and Harvard Medical School, Boston, Massachusetts, United States of America; 5 Celera Diagnostics, Alameda, California, United States of America; 6 Center for Comparative Genomics, University of Copenhagen, Copenhagen, Denmark; University of Aarhus, Denmark

## Abstract

Quantifying the distribution of fitness effects among newly arising mutations in the human genome is key to resolving important debates in medical and evolutionary genetics. Here, we present a method for inferring this distribution using Single Nucleotide Polymorphism (SNP) data from a population with non-stationary demographic history (such as that of modern humans). Application of our method to 47,576 coding SNPs found by direct resequencing of 11,404 protein coding-genes in 35 individuals (20 European Americans and 15 African Americans) allows us to assess the relative contribution of demographic and selective effects to patterning amino acid variation in the human genome. We find evidence of an ancient population expansion in the sample with African ancestry and a relatively recent bottleneck in the sample with European ancestry. After accounting for these demographic effects, we find strong evidence for great variability in the selective effects of new amino acid replacing mutations. In both populations, the patterns of variation are consistent with a leptokurtic distribution of selection coefficients (*e.g.*, gamma or log-normal) peaked near neutrality. Specifically, we predict 27–29% of amino acid changing (nonsynonymous) mutations are neutral or nearly neutral (|*s*|<0.01%), 30–42% are moderately deleterious (0.01%<|*s*|<1%), and nearly all the remainder are highly deleterious or lethal (|*s*|>1%). Our results are consistent with 10–20% of amino acid differences between humans and chimpanzees having been fixed by positive selection with the remainder of differences being neutral or nearly neutral. Our analysis also predicts that many of the alleles identified via whole-genome association mapping may be selectively neutral or (formerly) positively selected, implying that deleterious genetic variation affecting disease phenotype may be missed by this widely used approach for mapping genes underlying complex traits.

## Author Summary

Although mutations are known to cause varying degrees of harmful effects, it is difficult to quantify the distribution that best describes the variation of fitness effects of these mutations. Here we present a new method for inferring this distribution and inferring population history using Single Nucleotide Polymorphism (SNP) data from human populations. Using 47,576 SNPs discovered in 11,404 genes from sequencing 35 individuals (20 European Americans and 15 African Americans), we find evidence of an ancient population expansion in the sample with African ancestry and a relatively recent bottleneck in the sample with European ancestry. In both populations, the patterns of variation are consistent with a leptokurtic distribution of selection coefficients (*e.g.*, gamma or log-normal) peaked near neutrality. Specifically, we predict 27–29% of amino acid changing (nonsynonymous) mutations are neutral or nearly neutral, 30–42% are moderately deleterious, and nearly all the remainder are highly deleterious or lethal. Furthermore, we infer that 10–20% of amino acid differences between humans and chimpanzees were fixed by positive selection, with the remainder of differences being neutral or nearly neutral.

## Introduction

The distribution of fitness effects of newly arising mutations largely determines the importance of different evolutionary forces (*e.g.*, strong and weak selection, genetic drift, recombination, and bottlenecks) on the patterning the history of a population [1]. Quantifying this distribution could, therefore, inform debates in evolutionary and medical genetics including the advantages of sex and recombination [2], the applicability of neutral and nearly neutral theories of molecular evolution to population genetics [3],[4], and the importance of different population genetic theories of human complex disease [5]. Estimates for the proportion of mutations in the human genome that are neutral, deleterious, and nearly-neutral vary widely [6]–[11]. Many factors account for differences among estimates including quantity and quality of data, methodological approaches, and interpretation of results (including definitions of what is meant by deleterious, neutral, and nearly-neutral).

Two main approaches are used to estimate the distribution of fitness effects (DFE) of new mutations: direct estimates based on mutation accumulation (MA) experiments and indirect estimates based on population-genetic analyses of polymorphism and/or divergence data. MA experiments have generally found that the fitness distributions of new mutations are consistent with a leptokurtic (*i.e.*, highly peaked) DFE with a maximum at neutrality [12]–[15]. The precise functional form (*e.g.*, gamma, lognormal, etc.) remains unknown, however, since MA experiments are labor intensive and can only measure the fitness consequences of mutations of large effect. Another drawback is that MA cannot readily be applied to many species of interest (including humans) for both ethical and practical reasons.

Analyses of McDonald-Kreitman (MK) contingency tables (*i.e.*, comparison of the silent to replacement ratio of polymorphisms and fixed differences) have yielded various conclusions about the DFE, including that it may be normally distributed [16], nearly exponentially distributed [17], or gamma distributed with a shape parameter between 0.1 and 1 [18]. These tests have few degrees of freedom for model fitting and therefore have low power for distinguishing between alternative distributions. MK tables also ignore allele frequency information such as the relative proportion of low- and high-frequency SNPs.

Analyses that utilize the full distribution of allele frequencies (*i.e.*, the site-frequency spectrum) should provide better estimates of the DFE, although such methods would be very sensitive to the effects of demographic history and ascertainment biases in SNP discovery [19]–[21]. Furthermore, reliable outgroup data are needed in order to polarize SNPs (*i.e.*, distinguish between derived SNPs at low- and high-frequency), and multiple substitutions need to be considered carefully [22],[23]. The site-frequency spectrum has previously been used to estimate constant selection for non-stationary demographic models [19] and to estimate a DFE assuming the population under study is panmictic and constant in size [11], but neither study allowed for simultaneous inference of demography and a distribution of fitness effects. Recently, Keightley and Eyre-Walker [24] developed a method for simultaneous inference for demography and a distribution of fitness effects; however, their method is limited to very simple demographic scenarios (single population size change events) and was applied to a human dataset with a small number of loci without outgroup data. In this study, we describe a method to simultaneously infer both demography and the DFE from genome-wide polymorphism data, and describe the results of applying this method to a large polarized SNP dataset of 47,576 coding SNPs free of ascertainment bias, from two human populations. This approach enables us to estimate the DFE with more precision than previous approaches as well as explore various distributions that may underlie these effects and estimate the proportion of mutations fixed by adaptive evolution.

Briefly, we extend the Poisson Random Field approach outlined in Williamson *et al.* [19] for estimating demography and a constant selection coefficient to allow for inference of the DFE of newly arising mutations. This maximum likelihood approach uses putatively neutral synonymous SNPs to infer the demographic history of the population and then numerically solves for the transient distribution of allele frequencies for a given selective effect under that demographic model. We integrate these distributions over a range of possible fitness effects, weighted according to the candidate distribution, to find the maximum likelihood distribution of fitness effects. As in previous studies (*e.g.*, [1],[18],[24]), we focus on the DFE of deleterious nonsynonymous mutations. We assume *s* and 2*s* are the selective disadvantage of heterozygotes and homozygous mutants and infer the DFE based on the scaled selection parameter, γ = 2*N_e_s*. Our inference method only considers polymorphism data; however, once we have inferred the demographic and selective parameters, we can calculate the expected number of nonsynonymous fixed differences between humans and chimpanzees. Comparing our estimate to the number of observed differences offers a means of testing the predictions of our model.

We applied this method to a database of human autosomal coding SNPs ascertained without bias and for which a syntenic, informative chimpanzee outgroup nucleotide was available. SNPs were initially obtained by direct resequencing of exons from 20,362 putative genes in 15 African Americans and 20 European Americans. In total, 17.8 Mb of genic sequence (approximately 61% of autosomal RefSeq gene bases) containing 47,576 diallelic SNPs (25,145 synonymous and 22,431 nonsynonymous) from 11,404 genes met our bioinformatic criteria and were used in our population analysis.

## Results

### Numerical and Simulation Results To Test Performance of the Method

**Figure 1:**
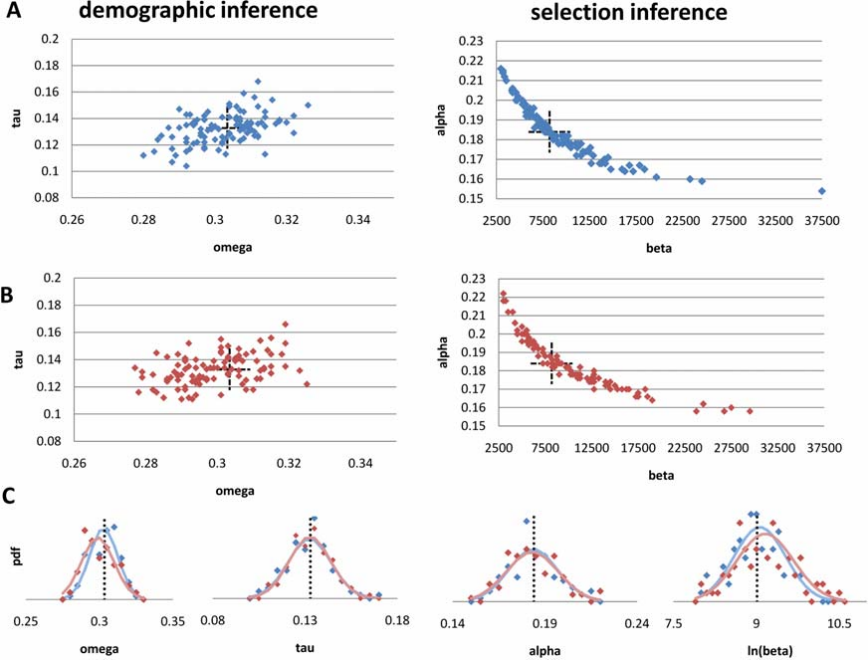
Simulation of demographic and selective parameter estimates with and without linkage. Simulation results for ML estimate of demographic and selective parameters assuming African American demography (τ = 0.1328, ω = 0.3034) and gamma distribution of fitness effects (α = 0.184, β = 8200). Sample sizes and mutation rates are the same as those in the African American data projected down to *N* = 24 chromosomes. Each panel represents 100 replicates; actual values shown with black dashed lines. (A) Simulations without linkage; each entry of the site-frequency spectrum is a Poisson variate drawn with the mean being that expected under the demographic model (synonymous sfs) or demography+selection model (nonsynonymous sfs). (B) Simulations with linkage; each entry calculated from a simulation of 11,404 genes, each with 7 linked exons (see Methods). (C) Distribution of inferred values for unlinked (blue) and linked (red) simulations.

The statistical method described below was implemented in an ANSI C computer program which estimates parameters of multi-epoch population size change models, and conditional on demographic parameters, estimates parameters of 13 different selection models (see Table 1). To test the accuracy of the program we simulated 100 datasets with and without linkage under the best-fit demographic model and gamma-distribution selection model inferred from the data (see Methods). The method was unbiased in estimating the demographic and selection parameters, and the amount of linkage in the dataset did not affect the accuracy or power of our inference appreciably (Figure 1).

**Table 1:** Maximum likelihood estimates of proposed distributions of deleterious fitness effects of new nonsynonymous mutations.

AFRICAN (observed fixed differences = 22,180)					
model	ΔLL	# fixed	df	distribution	MLE (95% C.I.)
neutral	8536.2	86,897	0	Pr(γ = 0) = 1	
fixed (pt mass)	2801.4	4,581	1	Pr(γ = k) = 1	k = −7.324 (−7.86, −6.81)
exponential	757.1	7,894	1	Pr(γ = −x) = EXP(λ)	λ = 0.0365 (0.0336, 0.0400)
neutral+lethal	136.0	28,016	1	Pr(γ = 0) = p^0^; Pr(γ = −∞) = 1−p^0^	p^0^ = 0.3224 (0.314, 0.331)
normal	225.9	65,078	2	Pr(γ = x) = NORM(μ,σ)	μ = −38.5 (−43.5, −34.0), σ = 28.6 (25.0, 32.5)
pt mass+lethal	44.0	17,878	2	Pr(γ = k) = p; Pr(γ = −∞) = 1−p	p = 0.372 (0.358, 0.387), k = −1.79 (−2.12, −1.45)
exponential+lethal	28.6	17,754	2	Pr(γ = −x) = p*EXP(λ); Pr(γ = −∞) = 1−p	λ = 0.373 (0.298, 0.464), p = 0.392 (0.375, 0.415)
exponential+neutral	7.0	22,133	2	Pr(γ = 0) = p^0^; Pr(γ = −x) = (1−p^0^)*EXP(λ)	λ = 0.0048 (0.0037, 0.0061), p^0^ = 0.245 (0.231, 0.256)
lognormal	5.1	19,812	2	Pr(γ = −x) = LOGNORM(μ, σ)	μ = 5.02 (4.55, 5.50), σ = 5.94 (5.18, 6.74)
gamma	3.7	20,113	2	Pr(γ = −x) = GAMMA(α, β)	α = 0.184 (0.158, 0.206), β = 8200 (3500, 20300)
neutral+pt mass+lethal	3.8	21,335	3	Pr(γ = 0) = p^0^; Pr(γ = k) = p; Pr(γ = −∞) = 1−p^0^−p	p^0^ = 0.245 (0.222, 0.266), p = 0.208 (0.176, 0.294), k = −13.3 (−8.8, −25.3)
neutral+gamma	2.9	20,758	3	Pr(γ = 0) = p^0^; Pr(γ = −x) = (1−p^0^)*GAMMA(α,β)	p^0^ = 0.148 (0.0, 0.235), α = 0.344 (0.178, 0.790), β = 1900 (280, 12300)
neutral+exponential+lethal	2.7	20,956	3	Pr(γ = 0) = p^0^; Pr(γ = −x) = (1−p^0^−p)*EXP(λ); Pr(γ = −∞) = p	λ = 0.02818 (0.0085, 0.072), p^0^ = 0.2176 (0.178, 0.245), p = 0.4525 (0.200, 0.548)
normal+lethal	–	24,300	3	Pr(γ = x) = p*NORM(μ,σ); Pr(γ = −∞) = 1−p	p = 0.428 (0.406, 0.458), μ = −4.44 (−5.40, −3.55), σ = 5.44 (4.4, 6.5)

EUROPEAN (observed fixed differences = 21,651)					
model	ΔLL	# fixed	df	distribution	MLE
neutral	5102.6	84,690	0	Pr(γ = 0) = 1	
fixed (pt mass)	1808.4	178	1	Pr(γ = k) = 1	k = −27.7
exponential	803.0	3,152	1	Pr(γ = −x) = EXP(λ)	λ = 0.00702
neutral+lethal	226.5	30,133	1	Pr(γ = 0) = p^0^; Pr(γ = −∞) = 1−p^0^	p^0^ = 0.3558
normal	164.0	233,700	2	Pr(γ = x) = NORM(μ,σ)	μ = −240, σ = 189
pt mass+lethal	85.5	9,209	2	Pr(γ = k) = p; Pr(γ = −∞) = 1−p	p = 0.430, k = −7.73
exponential+lethal	65.7	11,570	2	Pr(γ = −x) = p*EXP(λ); Pr(γ = −∞) = 1−p	λ = 0.0765, p = 0.45
exponential+neutral	3.3	20,747	2	Pr(γ = 0) = p^0^; Pr(γ = −x) = (1−p^0^)*EXP(λ)	λ = 0.0012, p^0^ = 0.24
lognormal	21.4	15,905	2	Pr(γ = −x) = LOGNORM(μ, σ)	μ = 5.95, σ = 5.22
gamma	15.1	16,705	2	Pr(γ = −x) = GAMMA(α, β)	α = 0.206, β = 15400
neutral+pt mass+lethal	5.1	21,681	3	Pr(γ = 0) = p^0^; Pr(γ = k) = p; Pr(γ = −∞) = 1−p^0^−p	p^0^ = 0.256, p = 0.35, k = −185
neutral+gamma	3.2	20,638	3	Pr(γ = 0) = p^0^; Pr(γ = −x) = (1−p^0^)*GAMMA(α,β)	p^0^ = 0.239, α = 1.02, β = 800
neutral+exponential+lethal	3.2	20,541	3	Pr(γ = 0) = p^0^; Pr(γ = −x) = (1−p^0^−p)*EXP(λ); Pr(γ = −∞) = p	λ = 0.00128, p^0^ = 0.2374, p = 0.026
normal+lethal	–	71,768	3	Pr(γ = x) = p*NORM(μ,σ); Pr(γ = −∞) = 1−p	p = 0.522, μ = −44.8, σ = 50.6

ML estimates and predicted number of human-chimp fixed differences under each model computed after applying demographic correction. Distributions are in terms of the scaled selection coefficient, γ = *2N_curr_s*, where *N_curr_* is 25,636 in African Americans and 52,907 in European Americans. *ΔLL* is the likelihood difference between the model and the overall best-fit model for the population; *# fixed* is the number of nonsynonymous fixed differences predicted by the model. Approximate 95% confidence intervals based on semi-parametric bootstrap are reported for African American parameter estimates.

### Inference of Demographic History

We find that a two-epoch instantaneous growth model provides a good fit to the synonymous site-frequency spectrum in the African American sample (χ^2^_obs_ = 40.3; *p*>0.05 based on coalescent simulations with recombination, see Figure S1, Figure S2, Figure S3 and Table S1). For the European American data, in contrast, growth or single bottleneck models were a poor fit (*p*<0.001). In order to even marginally fit the European American SFS data, a model with at least six parameters is necessary (*i.e.*, the “complex” bottleneck scenario; χ^2^_obs_ = 133.5; p∼0.01). The reason for this is that the European Americans show a large number of high-frequency derived SNPs even after correcting for multiple-hits using the method of Hernandez *et al*. [22]. This uptick in high frequency derived SNPs is also evident in the European American nonsynonymous site-frequency spectrum and because of the uncertainty in the cause of the departure from the model at synonymous sites, we focus much of our analyses on the African American data which showed no such bias.

### Distribution of Fitness Effects of Newly Arising Mutations

**Figure 2:**
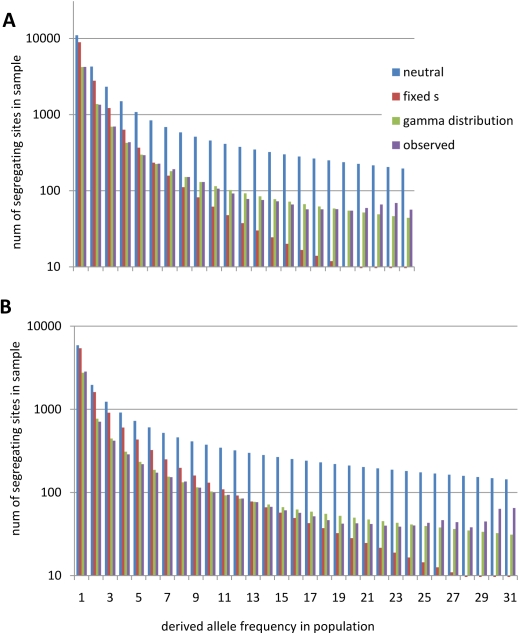
Observed and expected nonsynonymous site-frequency spectra after demographic correction. Expected site-frequency spectra under best-fit selection models after demographic correction. Note the logarithmic scale of the y-axis. (A) African American replacement SNPs versus expectation under neutrality, fixed selective effects, and gamma distribution of fitness effects. (B) European American replacement SNPs versus expectation under neutrality, fixed selective effects, and gamma distribution of fitness effects.

To infer the DFE at nonsynonymous sites, we analyzed the unfolded nonsynonymous site-frequency spectra using 13 different selection models (see Table 1 and Figure 2). After demographic correction (see Methods and Text S1), both populations showed less diversity than expected under strict neutrality at nonsynonymous sites, especially at high-frequency derived classes, indicative of purifying selection (Figure 2).

One-parameter models of selection, particularly those assigning a single selection coefficient to all nonsynonymous mutations, did a poor job of recovering the nonsynonymous site-frequency spectrum and predicting the number of observed nonsynonymous differences (Table 1). The best one-parameter model was a model of purifying selection which predicts 32.2% of amino acid sites are neutral, and 68.8% are strongly constrained. This model is 136 log-likelihood units below the maximum of more complex two- and three-parameter models, suggesting additional parameters provide highly significant improvements to the models.

Several two-parameter selection models—notably the gamma and lognormal—resulted in much better predictions (*ΔLL* = 3.7 and *ΔLL* = 5.1 below the maximum). All of these best-fit two-parameter selection models slightly under-predicted the number of observed nonsynonymous human-chimp differences which is consistent with the fixation of rare positively-selected mutations that were not incorporated into these models. Three-parameter selection models did not provide a measurably improved fit to the site-frequency spectrum except for the normal distribution model with a proportion of lethal site (normal+lethal, *ΔLL = 0*), which was the only model tested that allowed for weak positive selection on segregating variation.

**Figure 3:**
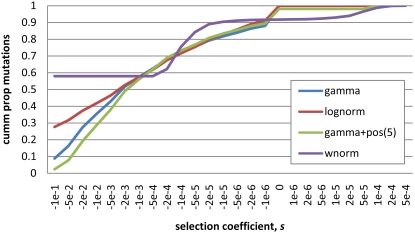
Cummulative proportion of nonsynonymous mutations with a selection coefficient less than *s*. Gamma and lognormal curves represent the best-fit gamma and lognormal models to the African American polymorphism data (Table 1). Gamma+pos and wnorm are the best-fit gamma distribution with positive selection at 2*N_e_s* = 5 and best-fit weighted normal model to the African American polymorphism+divergence data. All four distributions predict nearly identical site-frequency spectra that closely match the observed data. Left side are deleterious selection coefficients; right side are advantageous selection coefficients.

The best fitting gamma and lognormal distributions are both highly leptokurtic, with the bulk of mass centered on neutrality and a long negative tail extending into lethality. Our confidence in the proportion of nearly neutral mutations (|*s*|<0.0001) is tightly clustered around 28% in African Americans (27.3–29.0%, Tables 2 and 3). Our inferences are less tightly centered for more deleterious classes, as expected since the estimates of these classes are basically extrapolations of the distribution from the nearly-neutral data, since highly deleterious classes contribute little if any to observed polymorphism. Our confidence intervals incorporate both our demographic and selection parameter uncertainty, although they do critically depend on the selective effects following the gamma distribution. For example, the normal+lethal model also provides a good fit to the data but predicts somewhat different proportions of mutations in each selective class (Figure 3). This illustrates a general point that methods that make use of standing genetic variation to infer the strength of selection have little power to distinguish among models for the strength of highly deleterious mutations since these mutations contribute little to extant polymorphism. We note that in African Americans, the mean selection coefficient for newly arising mutations under the best-fit distributions is −0.058 for the lognormal and −0.029 for the gamma (95% C.I. = [−0.059, −0.018]), but the mean selection coefficient for mutations that are segregating (have two alleles in the sample) is less negative and nearly identical for the two distributions (−0.000136 for the lognormal and −0.000140 with 95% C.I. = [−0.000143, −0.000134]) for the gamma). Recently, Kryukov *et al.* [25] have estimated *s* for rare segregating human alleles, and their estimated range (−0.003– −0.001) is, encouragingly, between our estimate for new mutations and for segregating alleles.

**Table 2:** Distribution of fitness effects under various best-fit selective models.

Population ancestry	African	African	European	European	European	African	European	European
Demographic model	expansion	expansion	complex bottleneck	complex bottleneck	complex bottleneck	stationary	stationary	simple bottleneck
Selection model	gamma	lognormal	gamma	lognormal	exp+neut	gamma	gamma	gamma
*|s|*<0.00001	27.9%	28.4%	24.3%	24.6%	25.0%	22.2%	35.7%	23.5%
0.00001<|*s|*<0.0001	14.7%	14.3%	14.7%	15.7%	0.9%	48.2%	38.7%	16.1%
0.0001<|*s|*<0.01	21.9%	15.4%	23.1%	17.5%	53.7%	29.6%	25.4%	26.1%
*|s|*>0.01	35.5%	41.9%	37.9%	42.3%	20.5%	0.0%	0.2%	34.3%

Proportion of mutations falling into each selection interval for each population under each best-fit models. Demographic model parameters are listed in Table 3; selection model parameters are listed in Table 1 except for African American stationary gamma (α = 0.59, β = 37), and European American stationary gamma (α = 0.36, β = 120), and European American simple bottleneck gamma (α = 0.228, β = 5200).

**Table 3 pgen-1000083-t003:** Robustness of selective and demographic inference in African American dataset.

		full sfs		folded sfs	no singletons	silent site γ = −1
		MLE	95% C.I.	MLE	MLE	MLE
demographic parameters	Nanc	7778	7419–8143	7390	7847	10406
	Ncurr	25636	23863–27372	25221	22293	30778
	expansion	6809	6069–7862	7602	8083	5218
selection parameters	A	0.184	0.158–0.206	0.188	0.184	0.235
	β	8200	3500–20300	7600	12000	3150
*|s|*<0.0001		27.9%	27.3–29.0%	27.4%	25.3%	25.4%
0.0001<|*s|*<0.001		14.7%	12.8–16.9%	14.8%	13.3%	18.1%
0.001<|*s*|<0.01		21.9%	18.4–25.8%	22.3%	20.1%	28.8%
0.01<|*s*|		35.5%	29.2–40.7%	35.5%	41.2%	27.7%

Left column: MLE and approximate 95% confidence limits of demographic and selection parameter estimates for the full model. Center columns: MLE using folded site-frequency spectra (ΔLLsil = 1.89 and ΔLLrepl = 1.81 between folded MLE and full MLE) and using full site-frequency spectra excluding singletons (*i.e.*, derived frequency = 1 or n−1: ΔLLsil = 2.41 and ΔLLrepl = 0.0 between no-singleton MLE and full MLE). Right column: MLE assuming silent sites are under weak purifying selection (γ = −1).

Although selective inference is complicated by the complex demographic history of European Americans, we find no evidence that the DFE among new mutations differs between European and African Americans if we assume a gamma or lognormal distribution of fitness effects (Table 2; Figure S4). Other distributions provide a better fit to the European American data, but these all have a neutral point mass or a positive selection component which may lead to overfitting to the uptick of high-frequency derived SNPs observed in this population. We find it highly unlikely that the true DFE lacks moderately deleterious mutations, as is predicted by the best-fitting exponential+neutral or normal+gamma models inferred from the European American data (Table 2). We infer that European Americans have a similar mean selection coefficient for newly arising mutations as African Americans (−0.030 under the best-fit gamma model), but that the mean selection coefficient for segregating mutations in the sample is more negative in European Americans (−0.000344) than in African Americans (−0.000140). It is important to note that these estimates are likely associated with wide confidence intervals due to the complex bottleneck history in European-Americans (the large number of demographic parameters in the European-American model makes it computational infeasible to calculate the confidence intervals as we did for African-Americans).

Our estimates of the proportion of mutations that are strongly deleterious (|*s*|>1%), mildly deleterious (0.1%<|*s*|<1%), weakly deleterious (0.01%<|*s*|<0.1%), and nearly neutral (|*s*|<0.01%) are not affected by using the folded (*i.e.*, the distribution of minor allele frequency) vs. unfolded site-frequency spectrum or by removing singletons from the analysis (Table 3). This suggests that biases in SNP calling (which would predominantly affect singleton SNPs) and polarization of the ancestral state of mutations (which would only affect the unfolded SFS) have little influence on our inferences from the African American data. Furthermore, reasonable levels of weak, purifying selection at putatively neutral synonymous sites have only a modest affect on the inference of selection at nonsynonymous sites, although they can alter the demographic parameters significantly (Table 3).

### Distribution of Fitness Effects on Nonsynonymous SNPs and Fixed Differences

**Figure 4:**
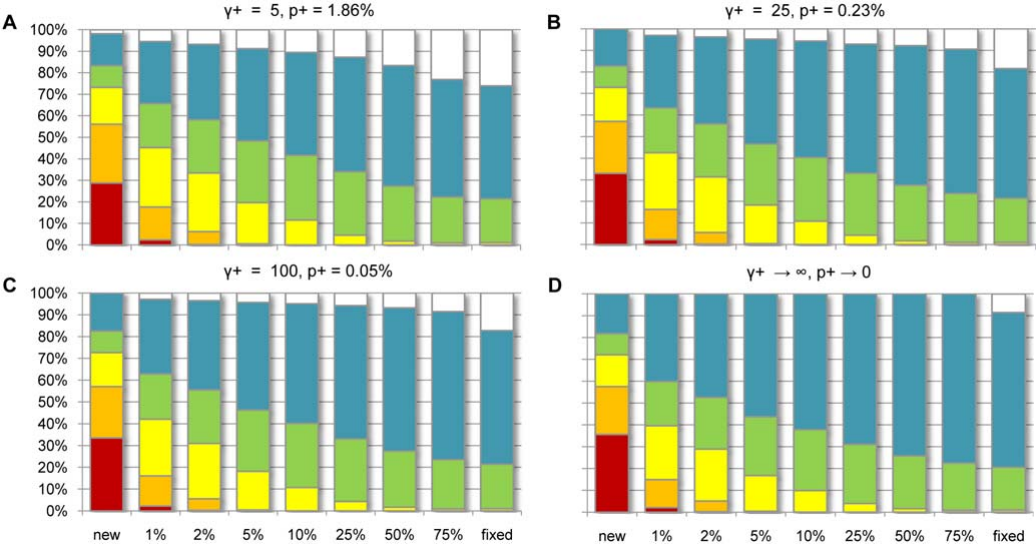
Inferred fitness effects of new, segregating, and fixed mutations in African-Americans. Estimated proportion of new nonsynonymous mutations (left column), SNPs (middle columns), and human-chimp fixed differences (right column) which are strongly deleterious (*s*<−10^−2^; red), moderately deleterious (−10^−2^<*s*<−10^−3^; orange), weakly deleterious (−10^−3^<s<−10^−4^; yellow), nearly neutral (−10^−4^<*s*<−10^−5^; green), neutral (−10^−5^<*s*<0; blue), and positively selected (white) in a sample of 100 chromosomes from a population under the best-fit expansion model of African American demography. (A–C) Proportions estimated by assuming all positively selected mutations have an effect of (A) γ+ = 5, (B) γ+ = 25, (C) γ+ = 100 and finding the MLE of the resulting three-parameter selection model (gamma distribution of deleterious fitness effects and a proportion (*p+*) of sites positively selected) to the African American polymorphism and divergence data. (D) Proportions estimated from the best-fit gamma distribution selection model (Table 1) in African Americans (equivalent to assuming positive selection is strong enough that positively selected mutants are never observed in the site-frequency spectrum). The resulting MLEs are (A) α = 0.228, β = 3100, *p+* = 0.0186; (B) α = 0.200, β = 5400, *p+* = 0.0023; (C) α = 0.196, β = 5850, *p+* = 0.0005; (D) α = 0.184, β = 8200, *p+*→0. Models (A–C) provided equally good fits to the polymorphism data, but they outperformed the best-fit gamma model and best-fit gamma+neutral model by 4.1, 3.5, 3.1 and 3.4, 2.8, and 2.4 LL units, respectively.

Based on our inference of the DFE of newly arising mutations, we can estimate the proportion of segregating SNPs or human-chimp fixed differences that fall within various selective ranges. Assuming the best-fit two-parameter gamma-distributed selection model, we estimate that around half of nonsynonymous mutations are strongly or mildly deleterious (|*s*|>0.1%). Because of the strength of purifying selection, however, we estimate that fewer than 0.5% of amino acid replacing SNPs segregating at any frequency above 5% have fitness effects this extreme (see orange bars in Figure 4). That is, most of the segregating variation above 5% frequency in the population is predicted to be nearly neutral (|*s*|<0.01%; Figure 4 green and blue bars) or weakly deleterious (0.01%<|*s*|<0.1%; Figure 4 yellow bars) with a higher proportion of neutral variation as the allele frequency increases. This culminates in a prediction that only 1% of nonsynonymous human-chimp differences are slightly deleterious (yellow bars), with the remainder being neutral or positively selected (Figure 4).

Our data do show significant evidence for positive selection having played an important role in patterning human-chimp amino acid divergence. All best-fit two- and three-parameter selection models which do not include positive selection (*i.e.*, all except Normal+lethal) underpredict the number of human-chimp nonsynonymous differences, consistent with a portion of differences being fixed through positive selection. For example, the expected number of nonsynonymous differences under the African American gamma model (20,113) is 9.3% less than what we observe (22,180; 95% C.L. = 5.4%, 12.8%). Modeling the DFE as a gamma function plus a positive selection component (Figure 4A–C) results in a significantly better fit to the nonsynonymous site-frequency spectrum than the best-fit gamma function (Δdf = 1; ΔLL = 4.1 for model in Figure 4B) or the best-fit gamma function with a neutral point mass (Δdf = 0; ΔLL = 3.3 for model Figure 4B). Furthermore, including positive selection of varying strength (from weak to moderate to strong) into the model explicitly (Figure 4A–C) increases our estimate of the proportion of differences fixed through adaptive evolution to around 20% instead of 9.3% but does not alter our deleterious mutation estimates appreciably. Based on these inferences, we expect that 10–20% of human-chimp differences were fixed through positive selection, depending on the exact nature of the DFE of beneficial mutations and the relative proportion of weakly versus strongly beneficial mutations. It is also important to note that if the strength of selection of beneficial mutations is very small (e.g., γ+≤5), then our approach predicts that more than 20% of human-chimp differences are adaptive and that appreciable levels of human segregating variation are subject to positive selection (Figure 4A).

### Concordance of PolyPhen Classifications and Inference of Selection

To further refine our estimates, we utilized PolyPhen [26],[27] to classify amino acid replacing SNPs as “benign”, “possibly damaging” or “probably damaging” based on site-specific sequence conservation among mammals as well as location in the three dimensional structure of the protein molecule (if known). (It is important to note that the term “damaging” used by PolyPhen is meant to reflect only that the mutation affects protein structure and not that the mutation results in loss or gain of function). After running PolyPhen, our dataset was reclassified into 15,916 benign, 4,199 possibly damaging, and 2,646 probably damaging SNPs, and we inferred the DFE for each class. As expected, the degree of purifying selection increases from benign to possibly damaging to probably damaging (Table 4). For possibly and probably damaging SNPs, the lognormal distribution was the best-fitting two-parameter model; for benign SNPs, the neutral+exponential distribution provided the best fit (Table 4). We estimate that approximately 5% of nonsynonymous differences classified by PolyPhen as benign were fixed through positive selection, while 27% and 35% of those classified as possibly and probably damaging were inferred to be fixed through positive selection. Furthermore, although the selection coefficient of a segregating benign mutation is less negative than that of a segregating damaging mutation, because there are so many more benign mutations and because they segregate at higher frequencies, the cumulative effect of benign mutations on fitness is greater than the cumulative effect of the rarer but more damaging mutations. Based on the best-fit models in Table 4 and the observed allele frequencies in each class, we predict the average fitness in the population is reduced by 13.4% due to weakly deleterious “benign” alleles but only by 4.3% and 3.2% for possibly and probably damaging alleles, respectively.

**Table 4:** Distribution of fitness effects in African Americans by PolyPhen class.

	benign	possibly damaging	probably damaging	combined
proportion of mutations	50.8%	25.7%	23.5%	
proportion of SNPs	72.7%	17.4%	9.9%	
proportion of fixed differences	82.4%	11.6%	6.0%	
best-fit selection model	neutral+exponential	lognormal	lognormal	gamma
ML parameters	λ = 0.0038, p^0^ = 0.409	μ = 4.69, σ = 2.83	μ = 5.78, σ = 2.87	α = 0.184, β = 8200
expected num of fixed differences	17,954	1,846	855	20,655
observed num of fixed differences	18,272	2,568	1,341	22,181
s<0.0001	42.0%	14.0%	7.4%	26.7%
0.0001<s<0.001	9.3%	25.5%	18.6%	15.7%
0.001<s<0.01	40.2%	31.3%	30.3%	35.6%
0.01<s	8.4%	29.2%	43.6%	22.0%
mean *s* (mutations)	−0.0030	−0.0087	−0.0265	−0.0294
mean *s* (polymorphisms)	−0.00013	−0.00027	−0.00040	−0.00014

Best-fit selection distribution for each PolyPhen class of mutations in African Americans and the proportion of new mutations in each PolyPhen class falling into each selection interval. Combined values are the sum of the best-fit values across classes. All selection inferences were run under the maximum likelihood African American expansion model.

## Discussion

This study represents the most comprehensive analysis to date using polymorphism frequencies to infer the DFE of newly arising mutations in the human genome. Several previous studies have suggested that the DFE is highly leptokurtic with a sharp peak around neutrality and a long tail extending into lethality [11],[18],[24]. Here, we find that such models provide a good fit to the data, but so does a mixture model with a Gaussian component centered on moderately deleterious mutations (∼45%) and 55% of mutations being lethal.

Our estimate that 27.3–29.0% of nonsynonymous mutations are neutral is remarkably consistent (although somewhat larger) than Eyre-Walker *et al*.'s [11] estimate of 23% (21–29%) inferred from a smaller dataset using folded frequency data and the hybrid approach of Yampolsky *et al.* [10] except that we expect a slightly larger proportion of mutations to be nearly neutral (Table 5). Using the folded site-frequency spectrum (*i.e.*, minor allele frequency distribution) or removing singleton SNPs had little effect on the predictions of the model (Table 3), suggesting that they are robust to errors in SNP calling, assembly, and alignment to the chimpanzee genome. Even if selection at synonymous sites were as strong as γ = −1 (stronger than current estimates in humans; *e.g.*, [28],[29]), the parameter estimates for the fitness distribution are within those estimated under the neutral assumption (Table 3). The demographic inference, however, changes if there is selection at silent sites—we infer a significantly larger population size undergoing an expansion event significantly more recently than we did under the assumption of synonymous sites being neutral (Table 3). Since γ = −1 is almost certainly an overestimate of selection on synonymous sites, and since the resulting model results in a poorer fit to both the synonymous and nonsynonymous site-frequency spectra (ΔLLsil = 14.4 and ΔLLrepl = 8.1 in African Americans), our inferences using γ = 0 at synonymous sites should be reasonable.

**Table 5:** A comparison of several recent estimates of the DFE at nonsynonymous sites in humans.

	this study		Eyre-Walker *et al.* [11]		Yampolsky *et al.* [10]
	African American (gamma distribution)	European American (gamma distribution)	basic gamma distribution	gamma with demographic correction	
*s*<0.00001	18%	15%	11%	10%	12%
0.00001<*s*<0.0001	10%	9%	8%	10%	14%
0.0001<*s*<0.01	37%	38%	37%	37%	49%
0.01<*s*	36%	38%	44%	29%	25%

Note that the selection intervals are those used by [10] and differ from those used in Tables 2–4.

It is important to note that our approach differs from some recent methods proposed for estimating the DFE on new mutations as well as some recent estimates of the DFE in humans (*e.g.,* [24]). That algorithm, as described and implemented, only considers a single size change event due to computational limitations of their matrix multiplication approach. Our method, on the other hand, uses an algorithm with running time independent of the number of size change events, so that is effectively no limit to the number of events that can be considered (*e.g.,* the population size can change every generation with essentially no added overhead). Our conclusions also differ from those of the previous group in that we find no significant difference in the DFE between African and European Americans, although we do find that alleles segregating in European Americans are on average more deleterious than those segregating in African Americans consistent with Lohmueller *et al*. [30]. Our comparison of observed versus predicted site-frequency spectra for silent sites suggests that our three-size-change model is a significantly better fit than the single-size-change model, and that the difference in DFEs between Europeans and African Americans reported by [24] may largely be a consequence of a poor fit of the single size change model to the European SFS data. That is, demography matters when estimating the parameters of the DFE as our and their analysis suggests and if the demographic model is mis-specified, estimates of the parameters in the DFE will be significantly biased.

Despite the large number of deleterious mutations that enter the population each generation, we estimate that the vast majority of common human genetic variation (e.g., SNPs at >5% derived allele frequency) is neutral or nearly neutral. We hold that these results have important implications for interpreting the outcome of whole genome association mapping studies that aim to identify alleles underlying common human diseases. Specifically, if chronic disease has a negative impact on Darwinian fitness, whole-genome association mapping approaches that survey common genetic variation for association with disease may be missing the most evolutionarily interesting and medically relevant alleles. The reason is that these assays have highest power to identify susceptibility alleles at moderate frequency, and are therefore biased towards finding evolutionarily neutral mutations (or formerly positively selected alleles). Re-sequencing in large samples of phenotypically extreme individuals, on the other hand, is much more likely to discovery rare, large-effect mutations that are predicted by our analysis (and others) to be deleterious. Population genetic theory also predicts that deleterious alleles will not spread widely from their geographic point of origin unless they stochastically drift in frequency due to founder effects or bottlenecks. Therefore, one would not expect, *a priori*, an association between a deleterious mutation and a disease phenotype to replicate across human populations even if the mutation has a significant attributable risk within a single population. Conversely, neutral polymorphisms (and formerly positively selected polymorphisms) that affect disease may likely replicate across human populations. These ascertainment biases in the evolutionary classes of alleles that can currently be associated with common human disease cloud our understanding of the relationship between chronic disease and Darwinian fitness.

Future work could extend this approach to allow for non-additive genetic effects and inference of more complex demographic models from joint site-frequency spectra. The method of Williamson *et al.* [31] of inferring selection and dominance could be incorporated into this model, although it is likely that the degree of dominance of new mutations follows a distribution whose shape may depend on the fitness effect of the mutation. Based on our results, it is unlikely that the synonymous and nonsynonymous site-frequency spectra contain enough information to allow for an inference of dominance parameters in addition to selective and demographic parameters. Like previous studies of the DFE in humans, our results should be interpreted as the distribution of heterozygote fitnesses over all genetic backgrounds [11].

A potential concern with our analysis is that we ignore linkage among selected alleles and among selected and neutral alleles in deriving predictions for the site-frequency spectrum. Ignoring linkage may bias our inference regarding the parameters of the DFE. It is important to note, however, the level of linkage in our dataset (15,681 nonsynonymous SNPs in African Americans spread over 11,404 genes and ∼100,000 exons) is slight and simulation results suggest that our parameter estimates and confidence intervals are robust to this complication (Figure 1).

A consequence of this DFE is that the molecular clock should be fairly insensitive to changes in *N*_e_ at nonsynonymous sites. Assuming mutations occur at a constant rate per generation, nearly neutral theory predicts that a selective regime that is gamma-distributed with a shape parameter α results in a rate of amino acid substitution proportional to *N*_e_^−α^ per generation [4],[32]. Since generation time is approximately proportional to *N*_e_^0.33^ in mammals [32], we expect amino acid replacing substitutions to fix at a rate proportional to ∼*N*_e_^0.15^ per year if α = 0.158–0.206 as we have inferred. This would explain why the observed relationship between population size and substitution rate is stronger at synonymous sites (which should fix at a rate proportional to *N*_e_^0.33^) than at nonsynonymous sites [3],[4],[33]. However, our estimate of α is sensitive to whether or not there is a point mass at neutrality in addition to a gamma distribution of fitness effects (Table 1).

Encouragingly, our frequency-based method of selective inference concurs with PolyPhen's fitness classification method which is based on conservation and physiochemical properties and does not utilize frequency information. Benign mutations are best described by a model with a significant neutral class (40.9%) and an exponentially-distributed deleterious class, whereas possibly and probably damaging mutations are best described by lognormal distribution with significant levels of deleterious mutation (Table 4), although these last two distributions were not significantly better fitting than the gamma distribution. Despite the heterogeneity of these distributions, the overall estimate of adaptive evolution is the same as that obtained from using a single gamma distribution—both models lead to a ∼10% underprediction in the number of human-chimp nonsynonymous differences. The proportionately higher levels of inferred adaptive evolution at possibly and probably damaging sites versus benign sites agrees with Kimura's [6] observation that stochastic loss of weak beneficial mutations results in mutations of intermediate effect being most likely to adaptively fix rather than those of small effect as predicted by Fisher [34].

In this investigation we did not look directly at the distribution of positively selected amino acid mutations owing to the difficulty of inferring the distribution when there are so many fewer positively selected mutations segregating than neutral or negatively selected ones. Nevertheless, we note that both African and European Americans exhibit about 10% more amino acid divergence than expected from best-fitting selective models that ignore positive selection. This suggests 10–20% of amino acid divergence is due to positive selection (Figure 4). This result are somewhat consistent with Fay *et al*.'s [8] estimate that 35% of human-chimp nonsynonymous fixed differences were adaptively driven, but contrast with recent calculations suggesting essentially none of these differences were driven by positive selection [35],[36]. We note that non-selective factors influencing fixation rates (such as linkage) should apply equally to synonymous sites and should not bias the number of nonsynonymous differences relative to the number of synonymous differences (divergence time was estimated from synonymous differences).

Despite the considerable dataset used in this study, our power to infer selective distributions was limited to two- and three-parameter models, some of which were indistinguishable. Although there are good theoretical and experimental reasons to prefer leptokurtic distributions like the gamma and the lognormal to fit the DFE (*e.g.*, [4],[37],[38]), even with a large, genome-wide resequencing dataset, site-frequency spectrum data alone are insufficient to rule out alternative models such as the normal+lethal (Gaussian shift) model. Previous studies have noted the lack of power to infer the tails of the fitness distribution from population genetic analyses, but we see that even distributions that differ significantly at intermediate frequencies can predict nearly identical site-frequency spectra (Figure 3). Furthermore, complex demography such as that found in our European American sample (see Text S1) can limit the power to infer selection even more. Lastly, it is important to note that we have not explicitly included migration in this analysis due to computational complexity. We hope to do so in future work, and while we suspect it will improve demographic inference, we do not expect it will greatly change our estimates of the DFE.

Although we encourage adding more genes to the dataset, such work is unlikely to increase the power of our analysis substantially. Deeper resequencing within humans, on the other hand, may likely yield improvements as estimates of rare allele frequencies are refined. Likewise, if the shape of the DFE is similar across species, analyzing the DFEs in species with various population sizes could be quite informative [1],[18],[24]. We note that when *N_e_* is very large, the assumption that synonymous sites are neutral may no longer be met [39]; however, our method is capable of jointly inferring the distribution of nearly neutral fitness effects and demography from the synonymous site-frequency spectrum prior to inferring the distribution of nonsynonymous fitness effects (as we did for Table 3). Independent estimates of the distribution of synonymous site fitness effects would be needed, however, to minimize the number of parameters being estimated from the site-frequency spectra on top of the demographic and nonsynonymous selective parameters.

## Methods

### Bioinformatic Pipeline

Resequencing was performed by Celera Genomics using PCR amplification of over 20,362 putative genes from the 2001 human genome in 19 African Americans and 20 European Americans (see [40] for a detailed description of this sequencing). These potential gene regions were included in our analysis if they could be uniquely mapped to hg18 in a region containing a refseq18 gene [41]. Refseq18 annotations were used to determine reading frame; regions that mapped to multiple Refseq genes were excluded if two or more of these genes were out-of-frame (89 kb of the 18.3 Mb of aligned regions). Furthermore, isPCR [42] was run on the 199k amplified primer pairs with perfect match = 15 and maximum product size = 800 bp. Regions with multiple isPCR hits (16.2 kb containing 76 SNPs) were removed from the data.

Finally, these high-quality amplified coding regions were included in the final analysis if they occurred in genomic regions corresponding to top-level syntenic or inverted chimp regions in net alignments between hg18 and panTro2 [42],[43]. In this way, chimp outgroup information could be used to polarize the SNPs. In total, we analyzed 17.8 Mb of aligned autosomal sequence containing 25,145 synonymous and 22,431 nonsynonymous SNPs and 56,555 synonymous and 36,138 nonsynonymous human-chimp differences.

### Inference of Demography

To control for the effect of demographic history on SNP frequencies, we used the method of Williamson *et al.* [19] to find the best-fit history of instantaneous population size changes to account for the frequency spectrum of synonymous SNPs assuming that such SNPs behave neutrally. To reduce any bias in this estimate caused by recent admixture, we removed from the analysis the four African Americans that showed high levels of European admixture. Since some SNPs did not amplify in all 15 non-admixed African American individuals or 20 (non-admixed) European American individuals, we performed a hypergeometric projection (gsl_ran_hypergeometric_pdf in Galassi *et al.* 2006) down to *N* = 24 chromosomes (in African Americans) and *N* = 32 chromosomes (in European Americans) [44]. SNPs where fewer than 80% of the chromosomes were sampled in a population or with some low quality base calls were excluded from the analysis for that population. After projection, the synonymous site frequency spectra contained 10746.1 and 8663.3 SNPs and the nonsynonymous site frequency spectra contained 7052.8 and 6273.0 SNPs for the African and European Americans, respectively (Table S2).

The putative ancestral state of each SNP was identified using the chimpanzee outgroup provided by the chimp genome (panTro2). Because mutation rates vary widely across the genome and are highly context dependent, we accounted for uncertainty in the ancestral state of each SNP following the method of Hernandez *et al.* [22]. Briefly, this method accounts for the probability of misidentifying the ancestral state of a SNP by modeling the observed frequency spectrum as a mixture of SNPs whose ancestral states were correctly identified and those that were misidentified under the context-dependent substitution process inferred by Hwang and Green [45] along the human lineage. This mixture model results in a system of equations whose unknown quantities represent the true frequency spectrum, which can readily be solved to correct for ancestral misidentification. This context-based correction is robust to the unequal distribution of CpG context between synonymous and nonsynonymous sites, a phenomenon that biases selection studies using nucleotide substitution matrices for multiple-hit correction [46].

Considering each population's corrected synonymous site-frequency spectrum separately, we used maximum likelihood to determine the best-fit demographic model. In both populations, a two-epoch model (two free parameters: timing and magnitude of size change) was a significantly better fit than a stationary model (Table S1). This two-epoch model was sufficient to account for the observed African American but not the observed European American synonymous site-frequency spectrum (Figure S1 and Figure S2). We optimized the parameters of the African American demographic model using a multinomial likelihood function conditioning on the number of SNPs observed in the sample (*i.e.*, each multinomial count is the proportion of SNPs in that frequency class multiplied by the total number of SNPs observed in the data) , and estimated the scaled human-chimp divergence time by finding the value that correctly predicted the number of observed synonymous human-chimp differences under this demographic model assuming a stationary model of chimp demography (with *N_e_* = human ancestral *N_e_*) and 300,000 generations since human-chimp divergence. We then set the European American ancestral population size and per-nucleotide mutation rate (μ) to that inferred from the African American data and fit a simple bottleneck (four free parameters: timing and magnitude of bottleneck; timing and magnitude of expansion) and a complex bottleneck (six free parameters: timing and magnitude of bottleneck; timing and magnitude of recovery; timing and magnitude of expansion) using a Poisson likelihood function and back-calculated European American *N_anc_* and μ using these parameters and a multinomial likelihood function. Although the maximum likelihood was nearly identical between the two bottleneck models and subsequent inferences of selection were robust to which of these two models we used (Table 2), the complex bottleneck provided a better goodness-of-fit, although it still failed to explain the excess of high-frequency derived SNPs in the sample. This excess may be caused by migration among Europeans and other populations or linkage to selected sites, although we cannot rule out uncorrected multiple hits or sequencing error. This excess is also apparent at European American nonsynonymous sites and is likely responsible for the overestimate in neutral and positively selected classes in some European American three-parameter selection distributions (Table 2).

### Inference of Distribution of Fitness Effects of Newly Arising Mutations

We use the distribution of sample frequencies among variable nucleotides in the coding gene alignments (*i.e.*, the site-frequency spectrum of coding SNPs) to infer the distribution of fitness effects among newly arising mutations. Our analytical approach makes use of standard Wright-Fisher population genetic theory within a Poisson Random Field setting [16], [19], [31], [47]–[49]. The assumptions of this model include independence among SNPs, genic selection, an underlying Poisson process governing mutations, and a piecewise constant population of large size amenable to modeling using diffusion approximations. The model we employ is an extension of Williamson *et al.* [19], where we present the relevant population and statistical inference theory for modeling genic selection in a population experiencing a recent size change. The key addition to our previous model is a distribution of fitness effects among new mutations. This amounts to modeling the components of the site-frequency spectrum—defined as (X_1_, X_2_, …, X*_n_*) where X*_i_* is the number of SNPs with *i* derived alleles in a population of *n*/2 individuals (*n* chromosomes)—as independent Poisson random variables with mean:
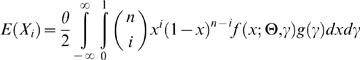
where *θ* is the genome-wide mutation rate, *x* represents the (unknown) population frequencies of mutations, *f*(*x*; Θ, *γ*) is the distribution of mutation frequencies given selection (γ  =  2*Ν_e_s*) and demographic history (Θ), and *g*(*γ*) is the distribution of fitness effects among new mutations. The synonymous mutation rate (*θ_S_*) was calculated from the number of synonymous segregating sites by the method of Williamson *et al*. [19]; the nonsynonymous mutation rate (*θ_N_*) was fixed as 2.5 × *θ_S_* [45]. Given the potentially different demographic histories of individuals with recent African and European ancestries, we model the two groups separately.

Although in theory γ = 2*N_e_s* can encompass any value from −∞ to ∞, we limited our integration of the distribution to −*N_e_* (corresponding to lethality) to −10^−6^ (corresponding to neutrality), plus a point-mass (usually at γ = 0) when appropriate. Only models incorporating a normal distribution were integrated above γ = 0. For distributions with a significant (>0.01%) mass between γ = −10^−6^ and 0, we added that mass to the weighting at γ = 0. Piecewise integrations were performed using midpnt, midinf, and qromo [50] with EPS = 2.5×10^−9^. Integration usually required evaluating the site-frequency spectrum at approximately 1,000 γ values. Each evaluation took around 1 sec and the result could be stored to facilitate the evaluation of subsequent distributions. Our algorithm, implemented in C, is available in the computer program **prfreq** available for download from the Bustamante lab website (<http://bustamantelab.cb.bscb.cornell.edu/software.shtml>).

To obtain approximate confidence intervals around the maximum likelihood estimates for each African American selective model, we generated 200 datasets from the observed African American synonymous and nonsynonymous site-frequency spectra by drawing a random Poisson variates for each entry of the site-frequency spectrum using the observed data as the mean for the variable. We optimized the demographic parameters for each synonymous dataset and then proceeded to optimize the selection parameters under that demographic model. These inferred parameters can be ordered to generate approximate 95% confidence limits that incorporate the uncertainty of both the demographic and selective parameter estimates. Exact 95% limits would require simulating datasets with linkage and simultaneously inferring the demographic and selection parameters. However, the level of linkage in our genome-wide dataset is slight and does not affect our estimates appreciably (Figure 1). Additionally, our algorithm is capable of jointly inferring the demographic and selection parameters, but computationally it is more demanding, and we find that the nonsynonymous site-frequency spectrum adds little power to our ability to infer demography (Figure S3).

### Inference of Mutational Effects across PolyPhen Classes

For each nonsynonymous SNP in our database, we sought a PolyPhen classification for every transcript in the human genome containing that SNP. Briefly, PolyPhen will classify a SNP as benign, possibly damaging, or probably damaging based on how conserved the site is across a multi-species alignment, functional annotation, and upon the structural consequences of the SNP [26],[27]. To assess evolutionary conservation, our sequences were aligned to those in the nrdb95 database (a union of the Swissprot, Swissnew, Trembl, Tremblnew, Genbank, PIR, Wormpep and PDB databases, with sequences of >95% similarity removed; [51]) using BLAST, and conservation was measured using PSIC. Functional annotations were taken from the UniProt database (http://www.pir.uniprot.org/), and structures were taken from PDB or PQS. Excluding SNPs that were unclassifiable or had different PolyPhen classifications for different transcripts, we retained 15,916 benign, 4,199 possibly damaging, and 2,646 probably damaging SNPs. The population-specific site-frequency spectrum for each mutational class was calculated as before, and selective inference was run assuming class-specific genome-wide mutation rates. To determine what proportion of new nonsynonymous mutations PolyPhen classifies as benign, possibly damaging, or probably damaging, we randomly mutated coding sequences from RefSeq 18 using a context-dependent substitution model [45]. We mutated 0.2% of the sites for a total of 46,492 nonsynonymous SNPs across 18,657 RefSeq transcripts that were then analyzed using PolyPhen. We found 59.5%, 21.2% and 19.3% of mutations were classified as benign, possibly damaging, and probably damaging, respectively, and used these proportions to calculate the class-specific genome-wide mutation rates. Finally, we also obtained PolyPhen classifications for each human-chimp fixed difference in our dataset.

## Supporting Information

Figure S1 Observed versus critical values of the χ^2^ goodness of fit test statistic using ms (Hudson 2002) with linkage to determine the test statistic distribution based on 1,000 runs of 22 chromosomes. (A) African expansion model: ms 24 22000 -t 269.4 -r 500 500 -en 0.0664 1 0.3034. (B) European simple bottleneck model: ms 32 22000 -t 308.9 -r 500 500 -en 0.0714 1 0.2629 -en 0.00725 1 0.1898. (C) European complex bottleneck model: ms 32 22000 -t 543.8 -r 500 500 -en 0.02505 1 0.1502 -en 0.02465 1 0.00495 -en 0.0027 1 0.1327.
(0.19 MB TIF)

Figure S2 Observed site frequency spectra at silent sites versus expected neutral site frequency spectra under best-fit demographic models. (A) African synonymous sites versus stationary expectation and best-fit expansion model. (B) European synonymous sites versus stationary expectation and best-fit expansion and bottleneck models.
(0.15 MB TIF)

Figure S3 Likelihood surface plots for demographic inference; τ is the timing of the expansion event in generations scaled by 2*N_curr_*, ω is the ratio of *N_anc_*/*N_curr_*. (A) African expansion model inferred solely from synonymous site frequency spectrum. (B) African expansion model inferred by simultaneous inference of demography and selection (assuming gamma distribution) with synonymous and replacement site frequency spectra.
(6.53 MB TIF)

Figure S4 Likelihood surface plots for selection distribution inference using a gamma model of selective effects: f(γ = 2*N_e_s*; α, β) = −γ^α−1^ [e^γ/β^]/[β^α^Г(α)] for γ<0. (A) African (*N_e_* = 25636): maxLL at α = 0.184, β = 8200. (B) European (*N_e_* = 52907): maxLL at α = 0.206, β = 15400.
(6.53 MB TIF)

Table S1 Summary of best-fit demographic models.
(0.04 MB DOC)

Table S2 Site-frequency spectra and human-chimp fixed difference counts used for inferences in this paper.
(0.09 MB DOC)

Text S1 Supplementary Material
(0.03 MB DOC)
