# Supplementary figures and images for: Assessing the Evolutionary Impact of Amino Acid Mutations in the Human Genome
Source: PLoS Genet. 2008 May 30;4(5):e1000083. doi: 10.1371/journal.pgen.1000083 (PMC2377339; doi:10.1371/journal.pgen.1000083)

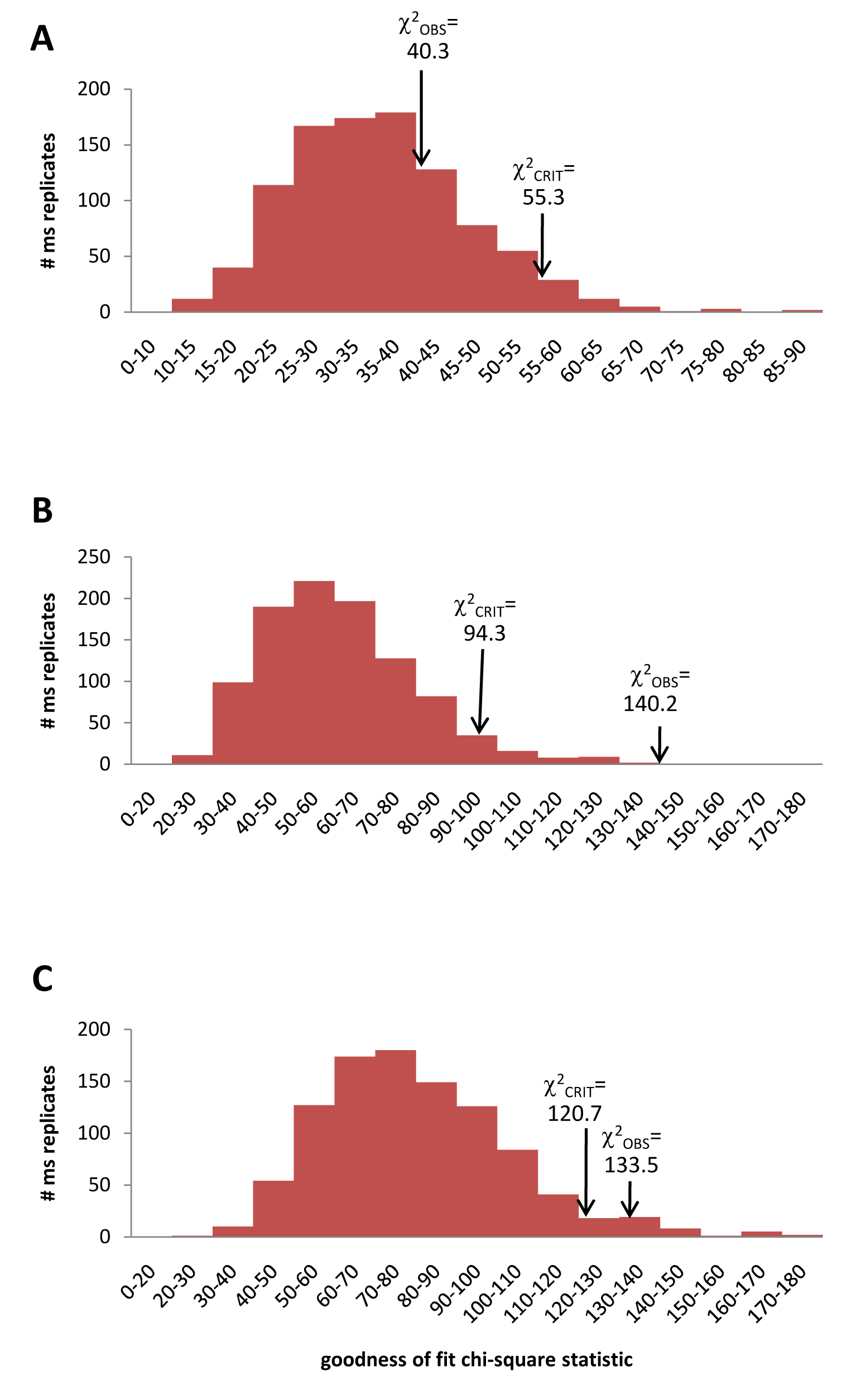

Supplement: Figure S1 — Observed versus critical values of the χ2 goodness of fit test statistic using ms (Hudson 2002) with linkage to determine the test statistic distribution based on 1,000 runs of 22 chromosomes. (A) African expansion model: ms 24 22000 -t 269.4 -r 500 500 -en 0.0664 1 0.3034. (B) European simple bottleneck model: ms 32 22000 -t 308.9 -r 500 500 -en 0.0714 1 0.2629 -en 0.00725 1 0.1898. (C) European complex bottleneck model: ms 32 22000 -t 543.8 -r 500 500 -en 0.02505 1 0.1502 -en 0.02465 1 0.00495 -en 0.0027 1 0.1327. (0.19 MB TIF) [file pgen.1000083.s001.tif]

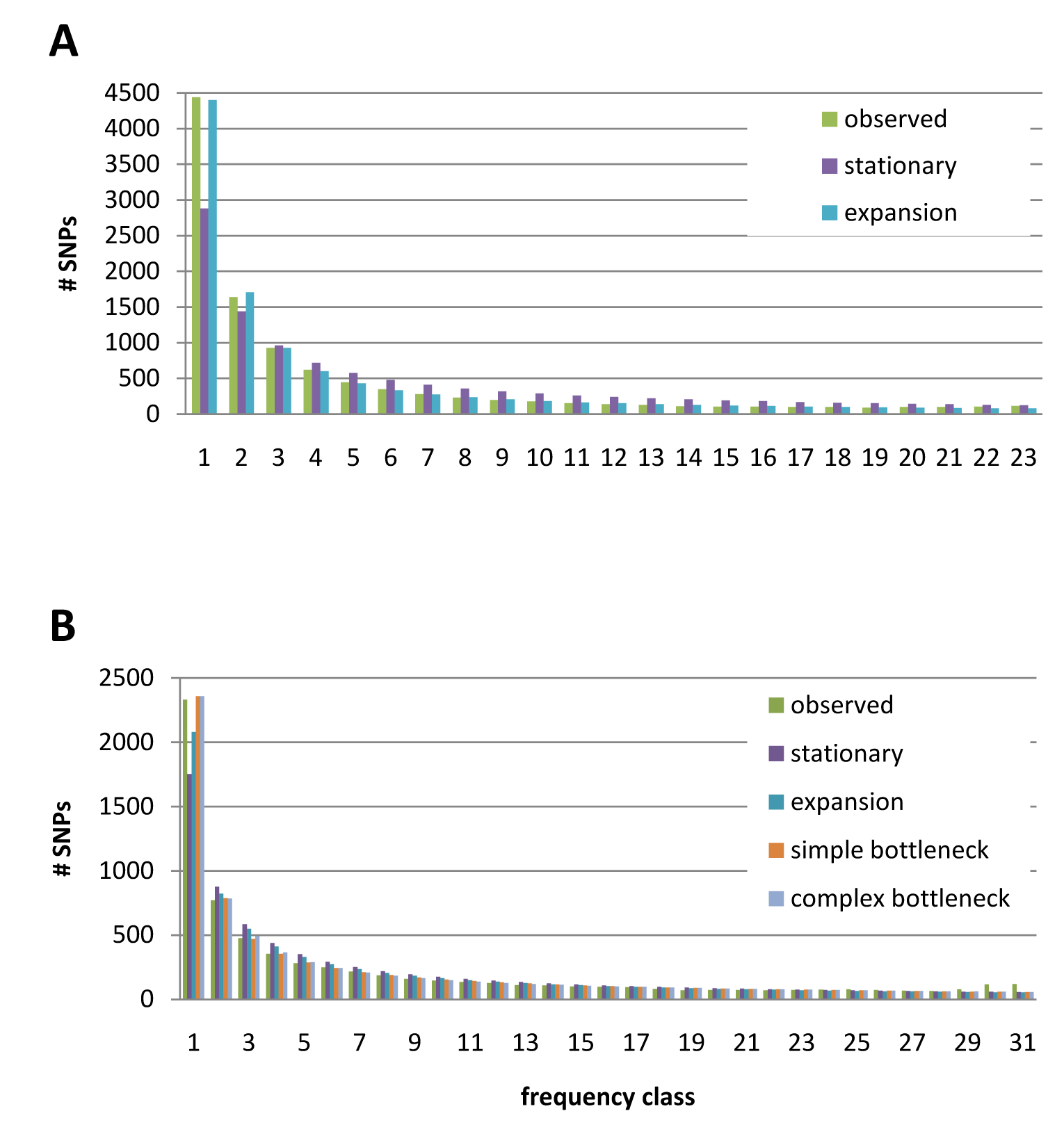

Supplement: Figure S2 — Observed site frequency spectra at silent sites versus expected neutral site frequency spectra under best-fit demographic models. (A) African synonymous sites versus stationary expectation and best-fit expansion model. (B) European synonymous sites versus stationary expectation and best-fit expansion and bottleneck models. (0.15 MB TIF) [file pgen.1000083.s002.tif]

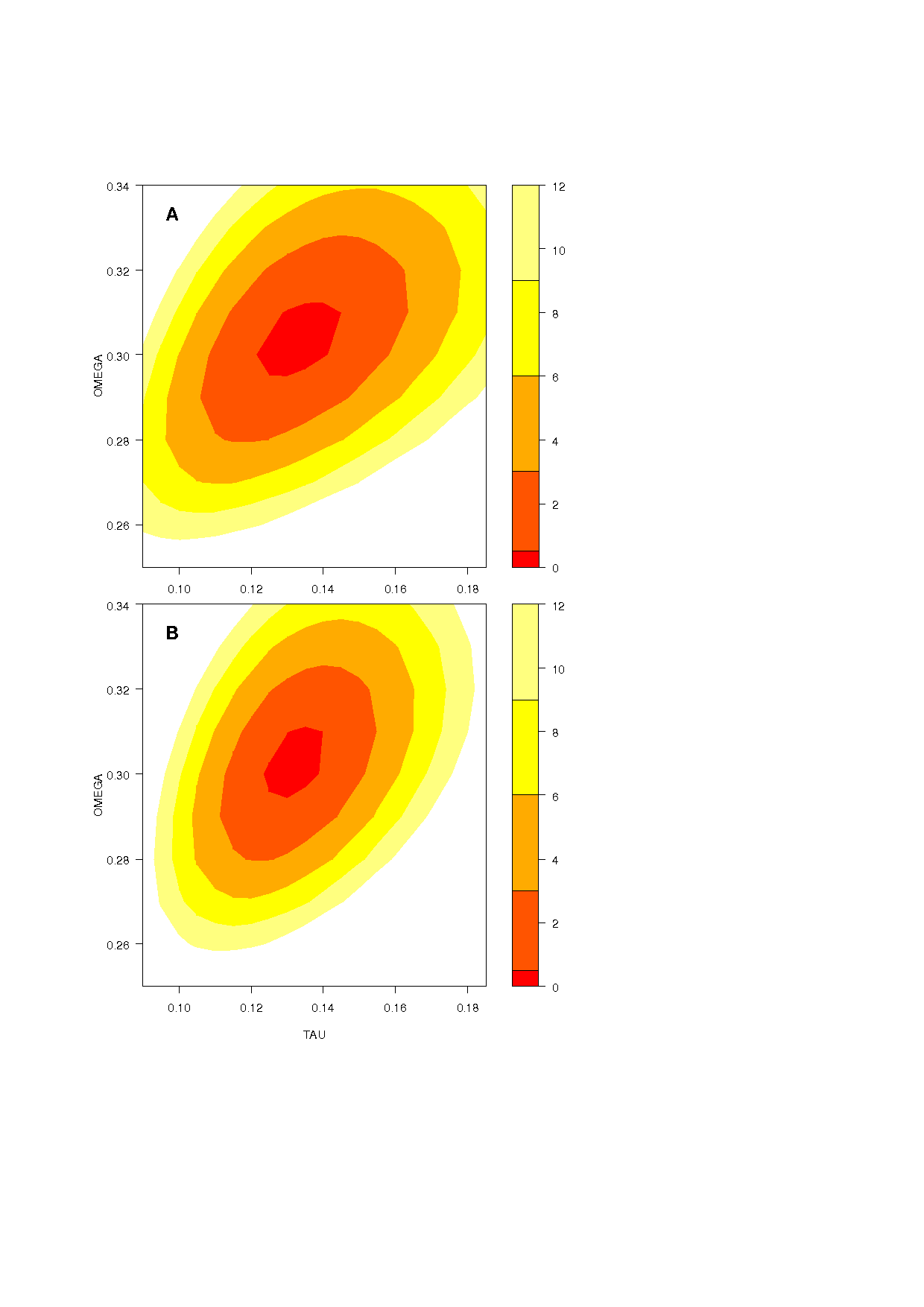

Supplement: Figure S3 — Likelihood surface plots for demographic inference; τ is the timing of the expansion event in generations scaled by 2Ncurr, ω is the ratio of Nanc/Ncurr. (A) African expansion model inferred solely from synonymous site frequency spectrum. (B) African expansion model inferred by simultaneous inference of demography and selection (assuming gamma distribution) with synonymous and replacement site frequency spectra. (6.53 MB TIF) [file pgen.1000083.s003.tif]

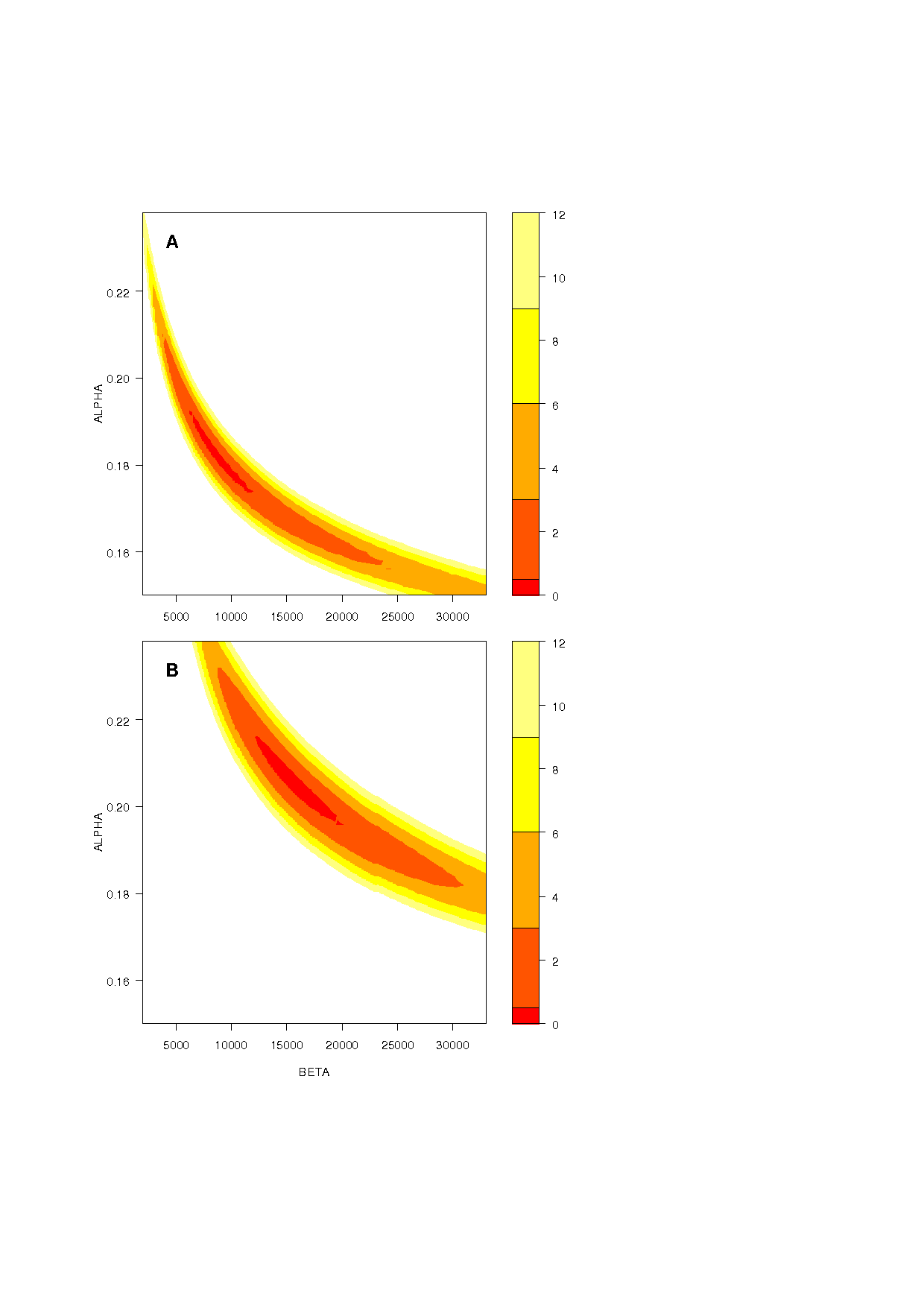

Supplement: Figure S4 — Likelihood surface plots for selection distribution inference using a gamma model of selective effects: f(γ = 2Nes; α, β) = −γα−1 [eγ/β]/[βαГ(α)] for γ<0. (A) African (Ne = 25636): maxLL at α = 0.184, β = 8200. (B) European (Ne = 52907): maxLL at α = 0.206, β = 15400. (6.53 MB TIF) [file pgen.1000083.s004.tif]
