# Supplementary material for: Assessing the Evolutionary Impact of Amino Acid Mutations in the Human Genome
Source: PLoS Genet. 2008 May 30;4(5):e1000083. doi: 10.1371/journal.pgen.1000083 (PMC2377339; doi:10.1371/journal.pgen.1000083)
Supplement: Table S1 — Summary of best-fit demographic models. (0.04 MB DOC) [file pgen.1000083.s005.doc]

**Table S1.** **Summary of best-fit demographic models.**

| *population* | *model* | *df* | Δ*LL* | *population sizes* | *expansion timings* | *bottleneck duration* |
| --- | --- | --- | --- | --- | --- | --- |
| African | stationary | 0 | 710.1 | *N_e_*=12420 |  |  |
| African | expansion | 2 | -- | *N_anc_*=7778, *N_curr_*=25636 | 6809 gen ago |  |
| European | stationary | 0 | 145.8 | *N_e_*=7754 |  |  |
| European | expansion | 2 | 47.9 | *N_anc_*=7175, *N_curr_*=153236 | 260 gen ago |  |
| European | bottleneck | 4 | 2.7 | *N_anc_*=7895, *N_btl_*=5699, *N_curr_*=30030 | 874 gen ago | 7703 gen |
| European | bottleneck + 2-step recovery | 6 | -- | *N_anc_*=7947, *N_btl_*=262, *N_rec_*=7019, *N_curr_*=52907 | 5217 gen ago, 576 gen ago | 84 gen |
