# Supplementary material for: Assessing the Evolutionary Impact of Amino Acid Mutations in the Human Genome
Source: PLoS Genet. 2008 May 30;4(5):e1000083. doi: 10.1371/journal.pgen.1000083 (PMC2377339; doi:10.1371/journal.pgen.1000083)
Supplement: Table S2 — Site-frequency spectra and human-chimp fixed difference counts used for inferences in this paper. (0.09 MB DOC) [file pgen.1000083.s006.doc]

**Table S2.** **Site-frequency spectra and human-chimp fixed difference counts used for inferences in this paper.**

| AFRICAN-AMERICAN | | | | | |  | EUROPEAN-AMERICAN | | |
| --- | --- | --- | --- | --- | --- | --- | --- | --- | --- |
| derived allele freqency | synonymous | nonsynonymous | benign | possibly damaging | probably damaging |  | derived allele freqency | synonymous | nonsynonymous |
| 1 | 4437.83 | 4228.09 | 2771.35 | 839.55 | 521.18 |  | 1 | 2329.14 | 2837.52 |
| 2 | 1640.09 | 1348.81 | 955.99 | 246.98 | 120.74 |  | 2 | 769.91 | 708.92 |
| 3 | 929.05 | 697.29 | 507.95 | 113.95 | 61.74 |  | 3 | 475.43 | 419.17 |
| 4 | 618.82 | 432.54 | 323.58 | 66.43 | 33.97 |  | 4 | 352.75 | 286.87 |
| 5 | 446.82 | 292.10 | 219.52 | 38.67 | 25.93 |  | 5 | 282.20 | 218.57 |
| 6 | 346.85 | 226.46 | 170.37 | 31.16 | 19.26 |  | 6 | 247.86 | 173.22 |
| 7 | 278.27 | 191.32 | 147.30 | 25.38 | 14.21 |  | 7 | 216.25 | 152.83 |
| 8 | 232.01 | 151.37 | 117.51 | 20.54 | 9.34 |  | 8 | 186.70 | 135.06 |
| 9 | 198.69 | 129.98 | 102.40 | 18.79 | 5.91 |  | 9 | 159.99 | 114.21 |
| 10 | 177.29 | 106.50 | 85.07 | 14.71 | 4.97 |  | 10 | 145.81 | 99.72 |
| 11 | 155.08 | 91.92 | 74.43 | 12.49 | 3.96 |  | 11 | 135.17 | 93.47 |
| 12 | 137.23 | 77.79 | 65.29 | 9.07 | 2.96 |  | 12 | 126.42 | 84.50 |
| 13 | 127.89 | 75.23 | 63.82 | 8.41 | 2.75 |  | 13 | 110.55 | 76.39 |
| 14 | 108.35 | 72.40 | 62.73 | 6.68 | 2.53 |  | 14 | 107.74 | 67.09 |
| 15 | 103.83 | 65.79 | 58.66 | 4.27 | 1.99 |  | 15 | 99.64 | 60.85 |
| 16 | 104.38 | 56.94 | 50.96 | 2.98 | 1.68 |  | 16 | 98.35 | 56.87 |
| 17 | 99.30 | 56.93 | 50.76 | 3.12 | 1.70 |  | 17 | 94.15 | 51.53 |
| 18 | 97.54 | 57.06 | 52.07 | 2.48 | 1.42 |  | 18 | 80.19 | 46.54 |
| 19 | 91.03 | 54.57 | 50.76 | 2.44 | 0.60 |  | 19 | 71.24 | 42.21 |
| 20 | 97.87 | 59.24 | 55.85 | 1.19 | 0.51 |  | 20 | 73.64 | 42.49 |
| 21 | 97.47 | 65.95 | 61.04 | 2.05 | 1.39 |  | 21 | 72.69 | 41.70 |
| 22 | 105.60 | 68.80 | 64.48 | 1.14 | 2.78 |  | 22 | 70.82 | 39.81 |
| 23 | 114.80 | 56.20 | 50.96 | 2.43 | 1.06 |  | 23 | 71.79 | 38.84 |
| FIXED | 34753.35 | 22180.26 | 18145.45 | 2443.00 | 1289.85 |  | 24 | 76.45 | 40.01 |
|  |  |  |  |  |  |  | 25 | 79.48 | 43.06 |
|  |  |  |  |  |  |  | 26 | 73.79 | 46.33 |
|  |  |  |  |  |  |  | 27 | 68.69 | 44.00 |
|  |  |  |  |  |  |  | 28 | 65.72 | 38.13 |
|  |  |  |  |  |  |  | 29 | 77.44 | 44.72 |
|  |  |  |  |  |  |  | 30 | 115.13 | 63.42 |
|  |  |  |  |  |  |  | 31 | 117.67 | 64.95 |
|  |  |  |  |  |  |  | FIXED | 33876.13 | 21650.62 |

Cell entries computed after quality control filters, sample size projection, polarization and multiple hits correction.
