# Supplementary material for: Assessing the Evolutionary Impact of Amino Acid Mutations in the Human Genome
Source: PLoS Genet. 2008 May 30;4(5):e1000083. doi: 10.1371/journal.pgen.1000083 (PMC2377339; doi:10.1371/journal.pgen.1000083)
Supplement: Text S1 — Supplementary Material (0.03 MB DOC) [file pgen.1000083.s007.doc]

**SUPPLEMENTAL INFORMATION**

**Counting the number of synonymous and non-synonymous human-chimp fixed differences**

After excluding regions of Celera amplicon coverage with poor human-chimp alignment or multiple RefSeq reading frames (see Methods), we categorized all differences between hg18 and panTro2 as synonymous or non-synonymous based on whether their change resulted in a change in amino acid. Changes to or from a stop codon were not included; nor did we include hg18-panTro2 differences occurring at polymorphic sites (i.e. those segregating in the Celera dataset). If two or more fixed differences occurred in a single codon, then we calculated all possible paths from the chimp codon to the human codon and chose that path that: first, minimized the number of nonsense (to/from stop codon) mutations; and secondly, minimized the number of nonsynonymous mutations. We then added the number of synonymous and nonsynonymous mutations in this path to the counts of synonymous and nonsynonymous fixed differences. From this number, we calculated the population-specific (African or European) fixed difference counts as follows: (1)since we excluded any segregating site which was did not have a high quality call in ≥80% of the individuals, we reduced our fixed difference tally by a similar proportion (6.2% in Africans and 4.0% in Europeans) to keep our estimates of Θ consistent; (2) we added to this any segregating site in the dataset with ≥80% call rate in the population if that population was monomorphic for the non-panTro2 allele (254 synonymous and 152 nonsynonymous differences in Africans and 732 synonymous and 481 nonsynonymous differences in Europeans); and (3) we added to this the portion of sites with ≥80% call rate and segregating with the non-panTro2 allele at high frequency in the population that would be expected to be lost if the population was projected down to 80% using the hypergeometric distribution (resulting in 215.0 synonymous and 119.3 nonsynonymous differences in Africans and 277.6 synonymous and 173.3 nonsynonymous differences in Europeans).

**Demographic inference of African- and European-American samples**

The overall levels of nucleotide diversity as well as the distribution of SNP frequencies, (*i.e*., the unfolded synonymous site frequency spectra) are strikingly different for non-admixed African Americans (“Africans”) and European-Americans (“Europeans”) (Fig. S2). Presumably, this is largely due the different demographic histories of these two groups. The site frequency spectrum of neither population is consistent with a stationary demographic history (P << 10^-10^ for both); instead, African synonymous site frequencies are best described by an ancient population expansion, while European frequencies show evidence of a more recent bottleneck followed by expansion (P = 10^-10^; see Table 1 for parameter estimates). Using a per-nucleotide per-generation mutation rate of 1.8×10^-8^, we estimate the current effective population size in Europeans to be twice that of Africans---Ne_EUR_ = 52,907 versus Ne_AFR_ = 25,636 (Table S1). The maximum likelihood estimates of the demographic events suggest a population increase from 7,778 to 25,636 occurred 6,809 generations ago in Africans, whereas Europeans experienced a short, sharp bottleneck 5,217 generations ago (from 7,947 to 262 individuals for 84 generations), followed by a population recovery to 7,109 individuals and eventually a population expansion to 52,907 individuals just 576 generations ago. The confidence intervals on the timing of these events are large and should be interpreted with caution, and the actual demographic histories are certainly more complex and include substructure and migration, which are not considered here. In both populations, the best fitting model underestimates the number of observed high-frequency derived SNPs, which could be a consequence of hidden substructure, linkage to selected sites, or incomplete correction for multiple hits in the observed data. Since the simple growth model did an excellent job of recovering the observed site frequency spectrum in Africans (Fig. S2), it should serve as reasonable corrections for the effect of demography on nonsynonymous sites when inferring the distribution of selective effects. For European SFS data, the correction is not as good

Our estimates of demography are largely consistent with those of Marth *et al.* (2004) which examined over 21,000 polymorphic sites and found that African-American diversity was best explained by a 1.8-fold expansion event 7,500 generations ago, quite close to our estimate of a 3.3-fold expansion 6,800 generations ago. Furthermore, this discrepancy is in the direction expected from our respective methods: Marth *et al.* did not attempt to control for recent admixture with Europeans, thus reducing the number of low-frequency SNPs in the sample relative to a pure African sample, which would reduce their estimated magnitude of the expansion. Marth *et al.* also concluded that a bottleneck model provided the best explanation of extant European diversity, although their prediction regarding the timing, duration and magnitude of this event are somewhat different (5-fold reduction for 500 generations followed by a 10-fold recovery 3,000 generations ago). We feel this concordance is remarkable given the low power associated with inferring the timing and duration of ancient bottlenecks and the high collinearity between estimates of bottlenecksize reduction and duration.

Nevertheless, our model predicts more private SNPs between Africans and Europeans than are observed and is at odds with current archeological and mtDNA evidence pointing to a more recent expansion and separation (probably ~60,000-80,000 years ago; see Mellars 2006 for a recent review). Presumably a low-level of historic migration between the populations (not included in our model) accounts for this discrepancy. Until datasets such as this are analyzed with more sophisticated models capable of inferring both size-changes and migration from multidimensional site frequency spectra, our preceding demographic estimates should be treated more as demographic corrections applied to polymorphism data than as precise conclusions regarding human population history. Timing and magnitude discrepancies aside, however, population-specific studies of human polymorphism consistently conclude that Africans have experienced a population expansion event while Europeans have undergone a bottleneck (Marth *et al.* 2004; Stajich and Hahn 2005; Plagnol *et al.*, 2006).

**Simulations to address effect of linkage on analysis**

The effect of linkage on demographic and selective inference was evaluated using forward population genetic simulations of a Wright-Fisher population. For each of the 100 replicate datasets, we simulated the evolution of a population with ancestral size, instantaneous size change, and current size identical to the best-fit expansion model for African demography (N_A_ = 7,778; N_C_ = 25,636; ω = 0.3034; τ = 0.1328) and selection operating at nonsynonymous sites. The *in silico* genome consisted of 11,404 genes with 7 exons of length 240bp and 2700bp introns. (These values were chosen to match the average number and length of exons and introns from all human genes in Refseq v.19). In our simulations, the codon structure was maintained, and new mutations were randomly introduced into the population at rate 0.0012 per basepair (as inferred from the synonymous SNPs in our dataset). Mutations that did not change the encoded amino acid (i.e. synonymous mutations) were assumed to be selectively neutral and therefore did not affect the fitness of the individual. Mutations that did change the encoded amino acid (nonsynonymous mutations), however, were assigned a selection coefficient that was drawn from a Gamma distribution with the shape and scale parameters inferred from our data (α=0.182, β=8400). We performed the simulations assuming a population scaled recombination rate equal to the population scaled mutation rate, estimated to be 0.0012 bp^-1^ from synonymous SNPs (an underestimate), as well as assuming completely unlinked SNPs for comparison (an overestimate). In each simulation, the population experienced an instantaneous growth event equivalent to the demographic history inferred from our African-American dataset. As the demographic event represents a change in effective population size, both the mutation rate and distribution of selective effects (which depend on effective size) were adjusted accordingly. We calculated the site-frequency spectrum with n = 24 chromosomes, estimated parameters of the demographic model, and conditional on estimates of the demographic model estimated the parameters of the selection model. The marginal and joint distributions of the estimated parameters are reported in figure 1. The site frequency spectra used for all the inferences in this paper are reported in Table S2.
